# Long-term culture of germ-free zebrafish using gamma-irradiated feeds

**DOI:** 10.1128/msystems.00353-26

**Published:** 2026-04-29

**Authors:** Lydia Okyere, Angela Di Fulvio, Christopher A. Gaulke

**Affiliations:** 1 Department of Pathobiology, University of Illinois Urbana-Champaign 612102, Urbana, Illinois, USA; 2 Department of Nuclear, Plasma, and Radiological Engineering, University of Illinois Urbana-Champaign 242173, Urbana, Illinois, USA; 3 Carl R. Woese Institute for Genomic Biology, University of Illinois Urbana-Champaign 124518, Urbana, Illinois, USA; 4 Personalized Nutrition Initiative, University of Illinois Urbana-Champaign 14589, Urbana, Illinois, USA; 5 Cancer Center at Illinois, University of Illinois Urbana-Champaign 612022, Urbana, Illinois, USA; 6 National Center for Supercomputing Applications, University of Illinois Urbana-Champaign 14589, Urbana, Illinois, USA; 7 Interdisciplinary Health Sciences Institute, University of Illinois Urbana-Champaign 612036, Urbana, Illinois, USA; University of Connecticut, Storrs, Connecticut, USA

**Keywords:** zebrafish, gut, microbiome, gnotobiotic, germ-free, host-microbe interactions

## Abstract

Host-associated microbiota play important roles in modulating key host functions, including digestion, nutrient acquisition, immunity, and xenobiotic metabolism. Disruption of these communities is linked to numerous diseases and health defects, though causal mechanisms underpinning these associations remain unclear in most cases. Gnotobiotic zebrafish provide a scalable low-cost *in vivo* model that is increasingly used to resolve causality in host-microbiota interactions. However, reliance on live diets and autoclaved diets limits the use of gnotobiotic zebrafish to early life stages where body systems and microbial communities are incompletely developed. As a result, many important host-microbiota interactions may be unable to be studied in this model system. Here, we tested a simple method for long-term husbandry of gnotobiotic zebrafish using gamma-irradiated chow diets and evaluated effects on growth, gene expression, and microbial community composition. In conventionally reared animals, gamma-irradiated diets did not affect growth or survival and only modestly impacted microbial community composition and diversity. In contrast, germ-free zebrafish maintained on sterile irradiated diets for 55 days post-fertilization were smaller, weighed less, and exhibited aberrant gene expression profiles relative to controls. These genes were enriched for pathways related to immune response, xenobiotic metabolism, organ development, liver function, and lipid metabolism, with many expression patterns linked to the abundance of specific microbial taxa. Together, these findings establish a practical protocol for long-term maintenance of gnotobiotic zebrafish and extend the utility of this model to study microbiome-dependent effects on host physiology and development beyond early larval stages of life.

**IMPORTANCE:**

While the gnotobiotic zebrafish have been a powerful model for interrogation of host-microbiota interactions, their use has been limited to early life stages due to complications of long-term husbandry. To address this limitation, we developed a simple protocol that enables rearing germ-free zebrafish well beyond larval stages. Germ-free fish exhibit physiological and developmental defects that mirror those described in mammalian counterparts supporting a conserved role for microbiota in vertebrate development and physiology. Our protocol provides a method to investigate microbial influences on adaptive immunity, metabolism, and chronic disease processes in zebrafish not possible with current methodologies. Given the rapid and simple methods for gnotobiotic derivation and the large number of transgenic animal lines available for zebrafish, we anticipate this model will accelerate mechanistic discovery of microbial impacts on host health.

## INTRODUCTION

The microbiome—the collection of microbes, genes, metabolic products, and ecological conditions at a location (1, 2)—is integral to animal health (3–7), influencing nutrient metabolism, immune development, pathogen resistance, and xenobiotic metabolism (4, 8, 9). While disruption of these communities is linked to diseases such as cancers (10), obesity (11), inflammatory bowel disease (12), and neurodegenerative disorders (13), the directionality of association and underlying molecular mechanisms are poorly defined. This knowledge gap limits translational impact and complicates the development of microbiome-modifying therapeutics.

Gnotobiotic animals, first developed in 1885, have been used to establish causal relationships between microbes and host physiology (14, 15), with gnotobiotic mice demonstrating microbiota-driven effects on immunity (16), brain function (17), metabolism (5), and development. However, the derivation of gnotobiotic mouse colonies is labor-intensive, time-consuming, and costly. To complement the mouse, several alternative model systems were developed and used to explore host-microbiome interactions (18). The gnotobiotic zebrafish was developed in 2004 (14) as a high-throughput model system cost-efficient model for mechanistic investigations of host-microbiota interactions (19). The transparent early life stages of zebrafish enable real-time investigation of microbial community assembly and responses to various stimuli in the gut (20, 21). External fertilization and facile gnotobiotic derivation allow the generation of thousands of gnotobiotic animals in less than a day at ~70× lower cost than mouse models. Gnotobiotic zebrafish have provided critical insights into microbial regulation of nutrient metabolism (14, 22), pancreatic β-cell development (23), intestinal epithelial proliferation (24), and innate immune system development (25). A major limitation of gnotobiotic zebrafish is that studies have been constrained to early life stages (<14 days post-fertilization). This limitation is largely due to reliance on live feeds (e.g., *Tetrahymena*) in conventional (CV) husbandry. While these diets provide rapid growth, they are difficult to make sterile (26, 27). Use of autoclaved diets (14, 28–31) is an alternative; however, adverse impacts of these diets noted in zebrafish (14) and germ-free (GF) mice (32, 33) raise questions about their safety and appropriateness for long-term culture. The lack of long-term gnotobiotic zebrafish husbandry protocols limits the utility of the model as larval fish (i) do not possess a functional adaptive immune system at this developmental stage (34, 35), (ii) have incompletely developed organ systems (36, 37), and (iii) house highly simplified gut microbiomes compared to adult fish and mammals (38).

The primary complication of long-term germ-free culture of zebrafish is lack of appropriate sterilized feeds. Existing gnotobiotic zebrafish feeding strategies—including UV-sterilized live feeds (27, 39), autoclaved diets, or no feeding—are suitable only for short-term studies (≤14 days post fertilization), and sterilization of live feeds remains laborious and contamination-prone for long-term husbandry (40). In mammals, autoclaved diets have been successfully used for gnotobiotic animal husbandry(41–43). However, epidermal degeneration has been reported in zebrafish fed an autoclaved diet in a prior study, although the relative contributions of diet processing and husbandry conditions, including water quality, remain unclear (14). Another widely used method of sterilization of foods is gamma irradiation, which damages DNA, thereby inactivating microorganisms (44). Currently, 40–50 kGy doses of gamma irradiation are the gold standard for food sterilization for gnotobiotic mice (45); however, it has not been investigated as a means for long-term culture of gnotobiotic zebrafish.

Here, we investigated the feasibility of gamma irradiation of feed for long-term husbandry of germ-free zebrafish. In addition to sterilization, gamma irradiation also damages dietary macro- and micronutrients leading to nutrient deficiencies, shifts in microbiome function, and immunomodulatory effects in the host (46, 47). Thus, we began by investigating the impact of feeding diets exposed to increasing doses of gamma-irradiated on zebrafish development and the microbiome structure. We then used these results to develop a long-term husbandry protocol for gnotobiotic zebrafish and evaluated the effects of microbiota and gnotobiotic derivation on gene expression and development. Our work demonstrates the feasibility of using gamma irradiation of dry diets for culture of gnotobiotic zebrafish far beyond previous standards and provides a foundational knowledge on the impact of the microbiome on zebrafish physiology.

## MATERIALS AND METHODS

### Animal experiments

#### Animal husbandry

Zebrafish (*Danio rerio* T5D strain) were housed on a 14:10 h light–dark cycle at 28°C and pH maintained between 7.0 and 7.3 and fed three times daily with GEMMA Micro 75 (Skretting, Westbrook, Maine) starting at 5 days post-fertilization (dpf).

#### Experiment 1—impact of gamma-irradiated diet consumption in microbiota-replete animals

Beginning at fertilization, conventionally raised zebrafish were housed in 75 cm^2^ polystyrene flasks (VWR; 5/flask), in six replicate flasks per group for 28 days. Fish were not treated with antimicrobials during this experiment. Water changes (50%) with zebrafish system water occurred every other day to maintain ammonia ≤0.5  ppm. This experiment was replicated twice (replicate 1, *N* = 67; replicate 2, *N* =70).

#### Experiment 2—long-term gnotobiotic culture of zebrafish

Germ-free (GF), conventionalized (CVZ), and conventional (CV) zebrafish were housed in eighteen 75 cm^2^ flasks (six per group; *N* = 5 fish per flask). An additional control group from the same spawn, colony controls (CCs), was used to evaluate growth and physiological consistency between experimental animals and animals raised under standard husbandry conditions. Colony controls were housed in a 2.8 L polycarbonate tank on a recirculating water system. Germ-free animals were maintained under sterile conditions after derivation; CVZ animals were derived germ-free but seeded with parental microbes immediately after derivation by exposure to parental tank water (i.e., spawn water); CV animals were housed like GF and CVZ but did not undergo germ-free derivation. All GF, CVZ, and CV groups received 50% embryo media (EM) (26) changes every other day and were all fed a supplemented 20 kGy irradiated diet, while the CC group was fed an unirradiated Gemma Micro 75 diet.

### Germ-free derivation

Germ-free T5D zebrafish were generated as previously described (26). Embryos collected within 1 h were transferred to sterile antibiotic embryo medium (ABEM) (26), which contained ampicillin (100 μg/mL), kanamycin (5 μg/mL), and amphotericin B (antifungal agent; 250 ng/mL) (Fisher Scientific, Waltham, MA, USA). Embryos were incubated at 28°C in ABEM for 6 h, rinsed (3×) with sterile EM (26), immersed in 0.1% povidone-iodine (broad spectrum antiseptic; Sigma-Aldrich, Saint Louis, MO, USA) for 2 m, rinsed (3×) with sterile EM, immersed in 0.003% sodium hypochlorite (disinfectant/antimicrobial) for 20 m, then rinsed (3×) with sterile EM. Sterilized embryos were transferred into 50 mL sterile EM in 75 cm^2^ flasks.

GF flasks were visually inspected with phase-contrast microscopy and EM aliquots were assessed for aerobic bacterial growth twice weekly at 28°C on Brain Heart Infusion (BHI) agar. For the final time point, aerobic and anaerobic growth of bacteria and fungi in EM was assessed on tryptic soy agar (TSA), BHI, and Sabouraud dextrose agar (SDA) as above. Gamma-irradiated diets were cultured on BHI agar, TSA, and SDA under aerobic and anaerobic conditions to confirm sterility. No microbial growth was detected in GF flasks (Fig. S1A and B) or irradiated diets (Fig. S1C and D).

### Gamma irradiation of diets

Diets were sterilized by gamma-irradiation (10, 20, 40, and 80 kGy) using a Gammacell Cobalt60 irradiator at the Nuclear Measurement Laboratory at the Department of Nuclear, Plasma, and Radiological Engineering, University of Illinois at Urbana-Champaign. The Gammacell 220 (48), manufactured by Nordion, is a self-contained Cobalt-60 irradiation unit consisting of sealed Co-60 sources enclosed within a lead shield, a cylindrical sample drawer, and a motorized drive mechanism that moves the drawer vertically along the source centerline. The irradiation cavity measures ~15 cm in diameter and 20 cm in height. The uniformity of the radiation field is ensured by the configuration of 48 double-sealed Co-60 source pencils, each 21.11 cm long, arranged in an annular formation around the chamber to provide a uniform gamma flux and minimize dose gradients. At the time of irradiation, the system delivered an average dose rate of approximately 2.1 kGy/h at the chamber center. The dose uniformity was validated using high-dose thermoluminescent dosimeters (TLDs) from Mirion, which confirmed a homogeneity of the irradiation field along the chamber diameter, with a measured dose variation of only ±3% (±1 SD). In the second experiment, we fed GF, CVZ, and CV fish with 20 kGy gamma-irradiated diets that had been supplemented with vitamins to mitigate potential micronutrient loss due to irradiation or reduction in vitamins synthesizing microbes. We supplemented irradiated Gemma Micro 75 with 5 mg thiamine HCl, 10 mg riboflavin, 10 mg calcium pantothenate, 0.6 mg D-biotin, 4 mg pyridoxine HCl, 1.5 mg folic acid, 200 mg inositol, 60 mg L-vitamin C-2-magnesium phosphate, 6.05 mg niacin, 50 mg α-vitamin E acetate, 4 mg vitamin K, 0.11 mg retinol acetate, 0.02 mg vitamin D, and 648.74 mg microcrystalline cellulose per gram feed (wt/wt) by spray-coating.

### Amplicon sequencing

DNA was extracted from pestle homogenized whole zebrafish using the DNeasy PowerSoil Kit (QIAGEN, Hilden, Germany) following the manufacturer’s protocol, with the addition of a 10-min incubation at 65°C prior to bead beating. The V4 hypervariable region of the bacterial 16S rRNA gene was amplified using the 515F and 806R barcoded primers (49). Library concentration was quantified with the Qubit DNA HS assay kit (Life Technologies, Carlsbad, CA, USA) and 200 ng of each prepared library was pooled and cleaned with the QIAquick PCR purification kit (QIAGEN). Libraries were sequenced on an Illumina MiSeq at the Roy J. Carver Biotechnology Center at University of Illinois at Urbana-Champaign. This generated ~10 million 300 bp paired-end reads (experiment 1, ~5.3 million; experiment 2, ~5 million).

### Microbial community analysis

#### Experiment 1

Raw paired sequence reads were filtered and trimmed using R (v4.5.0) and DADA2 (v1.20) with the following parameters: truncLen = c(240, 180), trimLeft = 7, maxEE = c(2,2), truncQ = 2, and rm.phix = TRUE. Forward and reverse reads were denoised, merged, and chimera filtered (consensus method). Taxonomy was assigned using the SILVA (v138.1) database (50, 51).

#### Experiment 2

Raw paired sequence reads were filtered and trimmed using R (v4.5.0) and DADA2 (v1.20) with the following parameters: truncLen = c(250, 230), maxN = 0, maxEE = c(2,2), truncQ = 2, and rm.phix = TRUE. Reads were denoised, merged, chimera filtered, and assigned taxonomy as above.

#### Diversity analysis and statistics

Experiment 1 and experiment 2 samples were rarefied to a depth of 7,000 and 70,000 reads, respectively. Samples with fewer reads than the rarefaction threshold were removed. Richness, Shannon entropy, and beta diversity (Jaccard) were calculated in R using the vegan package (v2.6-4). Principal component analysis (PCA) (stats v4.3.3) was visualized with ggplot2 (v3.5.0). Associations between microbiome beta diversity, feed, and replicate parameters were quantified with permutational multivariate analysis of variance (vegan::adonis2, 5,000 random permutations, *P* < 0.05). To assess the effects of experimental parameters on microbial diversity, generalized linear mixed models (GLMMs) were fitted using the glmmTMB package (v1.1.11).

### Transcriptome analysis

Total RNA was isolated from whole zebrafish juveniles with the AllPrep DNA/RNA Mini Kit (QIAGEN) following the manufacturer’s protocol. Prior to lysis, GF, CVZ, CV, and CC samples were flash-frozen in liquid nitrogen and homogenized using a sterile pestle. The concentration of RNA was assessed with the Qubit RNA HS assay kit (Life Technologies). RNA-Seq libraries were prepared with the Kapa Hyper Stranded mRNA library kit (Roche, Basel, Switzerland), pooled in equimolar concentrations, and sequenced on an Illumina NovaSeq X Plus (150 bp paired end).

FastQC (v0.11.9) assessed quality of paired-end reads before and after read trimming and filtering with Cutadapt (v2.6; -q 30, -l 50, --max-n=1). An additional nine bases were trimmed from the 5′ end and five bases from the 3′ end of each read to remove low-quality bases and residual adapter sequences (52). STAR (v2.7.10a) aligned reads to the zebrafish genome (GRCz11; Ensembl [53]) using default parameters (54). Differential expression was assessed with a likelihood ratio test in DESeq2 (v1.42.1) (55) and heatmaps and counts plots generated with ComplexHeatmap (v2.18.0) (56) and ggplot2 (v3.3.2), respectively. False discovery rate was controlled with the Benjamini–Hochberg method (FDR < 0.2). Gene set enrichment analysis (GSEA) was calculated for differentially expressed genes (DEGs; FDR < 0.1, log2 fold change > 0.5) gProfiler2 (v0.2.1) (57) and six databases: Gene Ontology–Biological Process (GO:BP), Gene Ontology–Molecular Function (GO:MF), Kyoto Encyclopedia of Genes and Genomes (KEGG), Reactome, and TRANScription FACtor (TF).

### Correlation analysis

Spearman’s rank correlation coefficient was calculated for all differentially abundant genes and bacteria (R stats v4.5.0). False discovery rate was controlled at FDR < 0.1 using the Benjamini–Hochberg procedure. Associations between bacterial abundance and gene expression were visualized with ComplexHeatmap.

## RESULTS

### Gamma-irradiation alters microbiome diversity but not zebrafish growth

**Fig 1:**
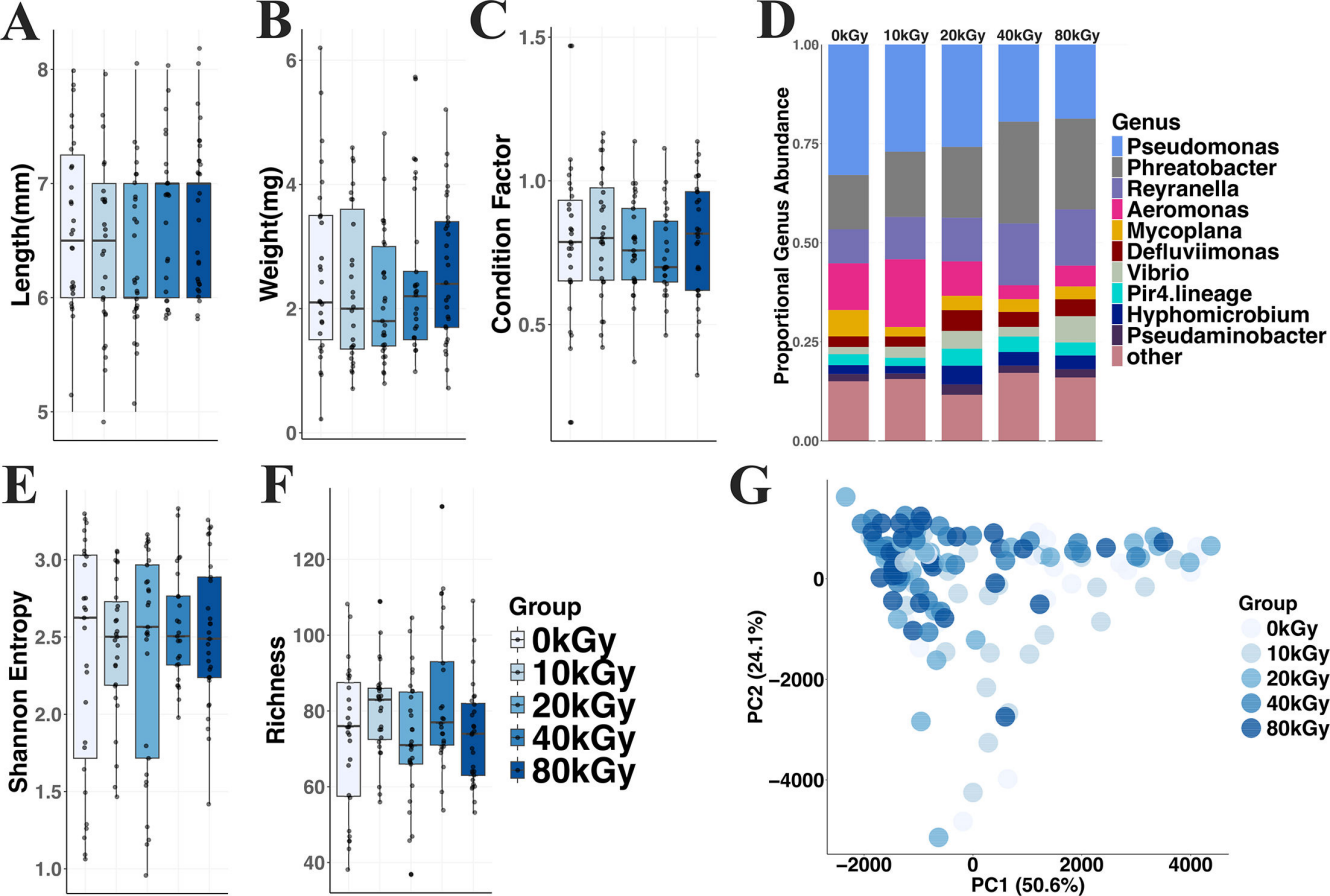
Gamma-irradiated diets do not affect conventionally reared zebrafish body metrics but alter microbiome composition. Boxplots show the effect of different diets on the (**A**) length, (**B**) weight, and (**C**) condition factor of zebrafish at 28 days post-fertilization (dpf). (**D**) The relative abundance of the top 10 microbial genera in the zebrafish. (**E**) Shannon entropy and (**F**) microbial richness in zebrafish fed the various diets. Individual points, jittered to reduce overplotting, represent data from individual fish. (**G**) Principal component analysis (PCA) ordination of microbial communities in zebrafish larvae fed gamma-irradiated diets compared to a non-irradiated diet (0 kGy, *n* = 27; 10 kGy, *n* = 27; 20 kGy, *n* = 29; 40 kGy, *n* = 25; and 80 kGy, *n* = 29), with colors denoting specific diets. Data are pooled from two independent experimental replicates.

To determine if diet gamma irradiation impacted zebrafish growth, we examined the weight and length of conventionally reared animals fed diets dosed with 0–80 kGy at 28 dpf. As expected, gamma irradiation resulted in sterilization of feed at all doses examined, while viable microbes were present in control feeds (Fig. S1C and D). Animals fed irradiated diets did not differ from controls in weight or length (Kruskal-Wallis; *P* > 0.05, Fig. 1A and B). Condition factor, an indicator of the fish health and growth similar to the body mass index (58), was also unchanged between groups (*P* > 0.05, Fig. 1C; Table S1). Relationships between length and weight, and length and condition factor were similar across all treatment groups (Fig. S1E and F). No differences in survival were noted between groups (Kruskal-Wallis; *H* = 4.0, *P* = 0.41). Together, these results suggest that feeding gamma-irradiated diets does not impact the growth or survival of larval zebrafish.

Since the microbiome is extensively linked to health, we asked if consumption of irradiated diets alters microbiome diversity. The microbiota of animals fed irradiated and control diets were compositionally similar (Fig. 1D). Neither microbial richness nor Shannon entropy was significantly impacted by irradiation (Kruskal-Wallis; *P* > 0.05; Fig. 1E and F). However, community beta-diversity was associated with dose (PERMANOVA; *R*^2^ = 0.07, *P* = 2.0 × 10^−4^; Fig. 1G). To determine which taxa may be impacted by different diets, we fit negative binomial GLMMs for each genus. We found 19 genera were significantly impacted by gamma irradiation, including several highly abundant taxa (Fig. S2A; FDR < 0.1). For example, *Phreatobacter* increased in animals fed 40 kGy (*z* = 3.7, *P* = 2.4 × 10^−4^) and 80 kGy (*z* = 2.9, *P* = 1.2 × 10^−3^) diets compared to controls while *Stenotrophomonas* was elevated in 80 kGy fed animals (*z* = 3.3, *P* = 1.1 × 10^−3^) (Fig. S2B). Conversely, *Aeromonas* abundance was decreased in 40 kGy (*z* = −3.2, *P* = 1.2 × 10^−3^) and 80 kGy (*z* = −2.0, *P* = 5.0 × 10^−2^) (Fig. S2B).

One possible explanation for the changes in microbiome composition is that irradiation killed microbes that normally would colonize the larval fish gut. If true, the compositions of microbiomes of control animals would be more similar to their diet than those fed irradiated diets. In contrast to the larval gut, feed microbiota was dominated by *Limosilactobacillus*, *Ligilactobacillus*, and *Candidatus* Competibacter (Fig. 1D; Fig. S2C) and did not differ across dose (ANOSIM: *R* = 0.11, *P* = 0.30). Zebrafish microbiomes had significantly greater richness (Kruskal-Wallis; *H* = 5.7, *P* = 1.7 × 10^−2^) and lower Shannon entropy (Kruskal-Wallis; *H* = 15.1, *P* = 9.9 × 10^−5^) compared to diet (Fig. S2D and E). Microbial community diversity was associated with sample type (feed vs fish; PERMANOVA; *R*^2^ = 0.16*, P =* 2 × 10^−4^; Fig. S2F). However, control diet microbiota were not more similar to their feed when compared to irradiated diet microbiota (*P* > 0.05, Fig. S2G). Fourteen ASVs were shared between feed and fish samples, representing a diverse set of taxa including lactic acid bacteria, *Ottowia*, and *Streptococcus* (Fig. S2H). Thirteen of these ASVs were abundant in feed but occurred at low relative abundance or were undetectable in fish across treatments, suggesting limited colonization of fish. In contrast, *Aeromonas* (ASV4) was consistently detected at higher relative abundance in fish despite being rare or absent in feed. No shared ASV showed patterns consistent with enrichment in fish fed the non-irradiated (0 kGy) diet, indicating limited evidence for diet-driven establishment of these taxa in the fish microbiome. Together, these data suggest that alterations in zebrafish microbial communities are likely driven by factors other than direct establishment of microbes originating from feed.

### Gamma irradiation of diet enables long-term husbandry of germ-free zebrafish

**Fig 2:**
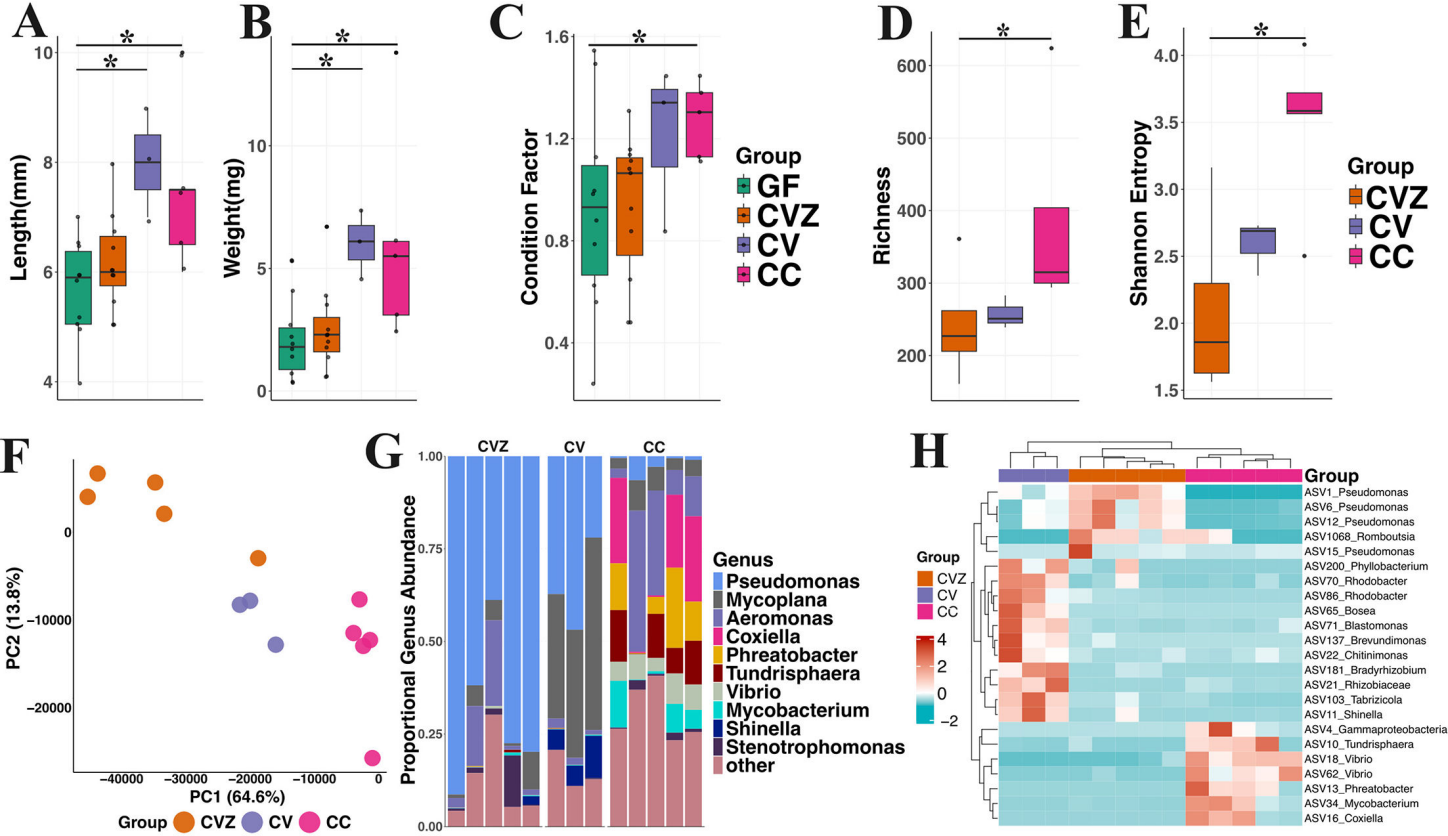
Zebrafish growth metrics and microbiome composition differ across experimental groups. Boxplots depict the (**A**) length, (**B**) weight, and (**C**) condition factor of GF (*n* = 5), CVZ (*n* = 5), CV (*n* = 3), and CC (*n* = 5) zebrafish at 55 days post-fertilization (dpf). (**D**) Microbial richness and (**E**) Shannon entropy boxplots for zebrafish in the CVZ, CV, and CC groups. Individual points, jittered to reduce overplotting, represent data from individual fish. (**F**) Principal component analysis (PCA) ordination illustrating the clustering of microbial communities among CVZ, CV, and CC zebrafish, with colored dots representing different groups. (**G**) Relative abundance of the top 10 microbial genera across CVZ, CV, and CC zebrafish. (**H**) Heatmap showing differentially abundant taxa among CVZ fish, CV fish, and CC fish. Each column represents an individual sample, and each row represents a taxon identified as significantly different among groups. Color intensity reflects *Z*-score normalized abundance across samples, with red indicating higher abundance and blue indicating lower abundance. Only taxa with *P* < 0.005 and *q* < 0.2 were included.

Next, we examined the feasibility of using gamma-irradiated diets to rear germ-free zebrafish past the larval stage (~2 weeks). We selected 8 weeks as a terminal time point as many aspects of social behaviors (59), immune function (26), and gastrointestinal system anatomy and function (60), not yet fully matured at 2 weeks post-fertilization, are completed or advanced significantly at this time. We began by examining differences in growth in GF, CVZ, and conventional CV animals reared on 20 kGy diets for ~8 weeks (55 dpf), as well as CC reared under standard husbandry conditions (see Materials and Methods). Weight (GLMM; CVZ: *z* = 0.46, *P* = 0.65; CV: *z* = 2.74, *P* = 6.0 × 10^−3^; and CC: *z* = 3.42 *P* = 6.2 × 10^−4^) and length (CVZ: *z* = 1.11, *P* = 0.27; CV: *z* = 3.65, *P* = 2.7 × 10^−4^; and CC: *z* = 3.4, *P* = 6.0 × 10^−4^) were significantly increased in CV and CC compared to GF, while the CVZ group did not differ significantly. Condition factor did not vary across group (*P*>0.05; Fig. 2A through C; Table S2). Length-weight and length-condition factor relationships were continuous across the full-length range in all groups, with no evidence of group-specific clustering (Fig. S3A and B).

Since husbandry and derivation conditions differed between groups, we examined how microbial diversity and composition differed across groups. Richness was increased in CV (*z* = 6.16, *P* = 0.83) and CC animals (CC: *z* = 2.58, *P* = 1.0 × 10^−2^) compared to CVZ (Fig. 2D). Shannon entropy increased only in CC animals (*P* = 0.04; Fig. 2E). Community beta diversity is also associated with group (*R*^2^ = 0.48, *P* = 4 × 10^−4^) (Fig. 2F). Consistently, differences were also noted in genera (Fig. 2G; Table S3) and ASV abundances (Fig. 2H; Table S4). Highly abundant ASVs corresponding to the genus *Pseudomonas* (ASV1, ASV6, and ASV12) and *Shinella* (ASV11) were elevated in CVZ fish compared to CC animals, while *Phreatobacter* (ASV13 and ASV66), *Vibrio* (ASV18), and *Coxiella* (ASV16) were depleted in CVZ and CV (Table S4). Together, these data indicate that germ-free derivation or gamma irradiation likely influences microbial community structure.

### Germ-free animals manifest unique gene expression profiles

**Fig 3:**
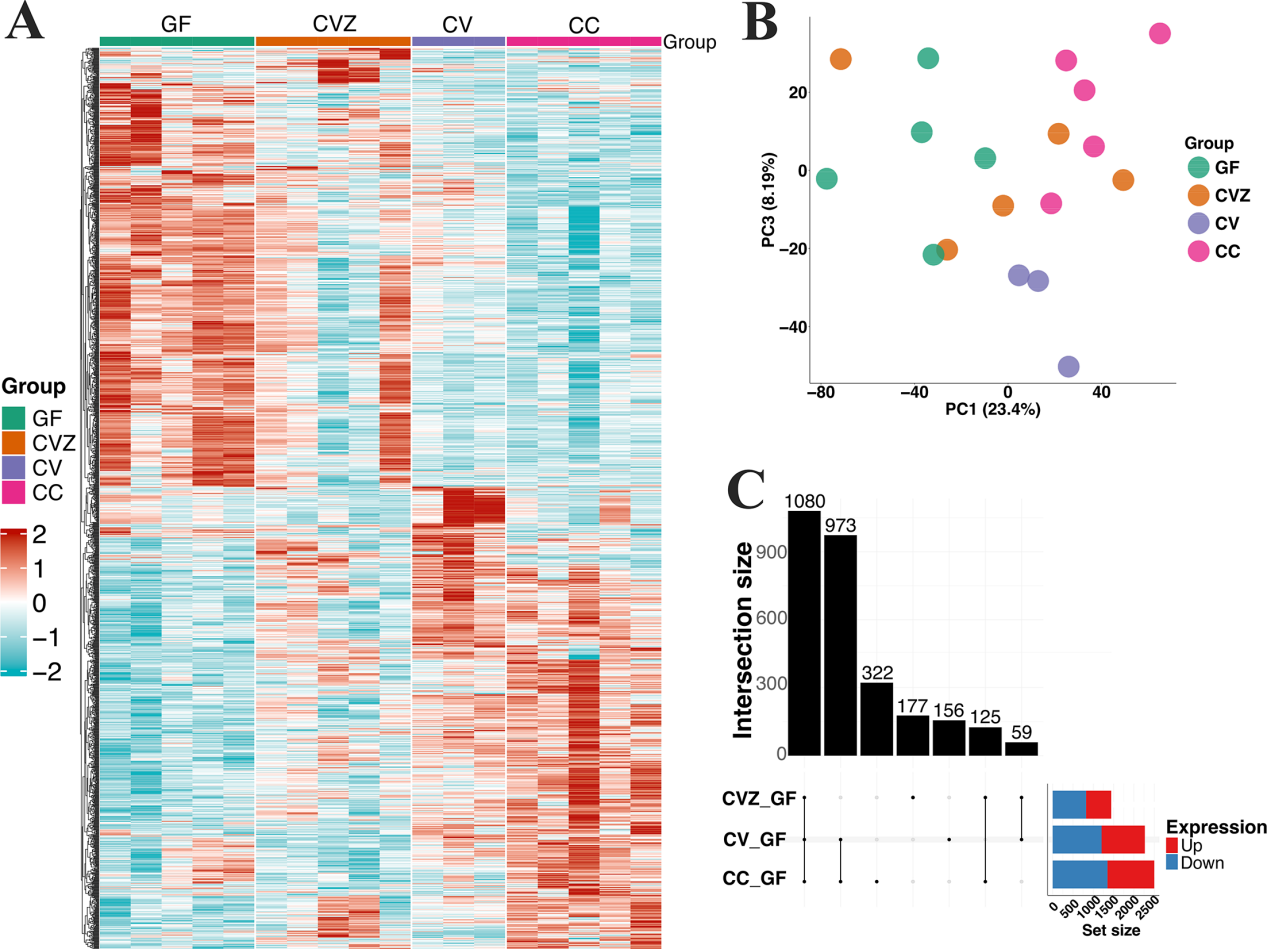
Germ-free zebrafish exhibit distinct gene expression profiles compared to microbe-colonized animals. (**A**) Heatmap showing the differentially expressed genes in GF, CVZ, CV, and CC fish. The different groups are indicated by colored bars on the top. (**B**) Principal component analysis (PCA) plot showing the clustering of expressed genes in GF, CVZ, CV, and CC fish. (**C**) Upset plot showing the intersection of shared and unique genes of CV, CVZ, and CC fish in reference to germ-free fish (GF).

To investigate the impact of microbial communities on host physiology, we conducted whole animal RNA-Seq of individual GF, CVZ, CV, and CC zebrafish (*N* = 5, 5, 3, and 5 per group, respectively). In total, 2,615 DEGs (FDR = 0.1) were detected between groups (Fig. 3A; Table S5) with group explaining ~27% of gene expression variance (*R*^2^ = 0.27, *P* = 6.0 × 10^−3^; Fig. 3B). When compared to GF, 1,441; 2,268; and 2,500 genes were differentially expressed in CVZ, CV, and CC groups, respectively (Fig. 3C). Of these, 1,080 genes showed consistent directional changes across groups, while additional subsets were shared between CC and CV or unique to CC.

GF zebrafish exhibited reduced expression of genes involved in intestinal development and immune signaling, including *vil1*, *il1b*, *cd36*, *ncf1*, *gata3*, *stat4*, *mhc2daa*, and *zbtb7b*. Genes associated with lipid and bile acid metabolism (*fabp2*, *akr1b1.2*, *ugt2a6*, *apoa1a*, *cyp27a2*, and *cyp3a65)* were also downregulated, consistent with microbial modulation of lipid and bile acid metabolism in vertebrates (61, 62). Consistent with prior work in germ-free, genes involved in recognition of bacterial products (e.g., *cxcl8a* and *ly97.3*) previously shown to be decreased in germ-free zebrafish (63–65) were also reduced in our data. Together, these gene-level patterns motivated subsequent pathway-level analyses.

**Fig 4:**
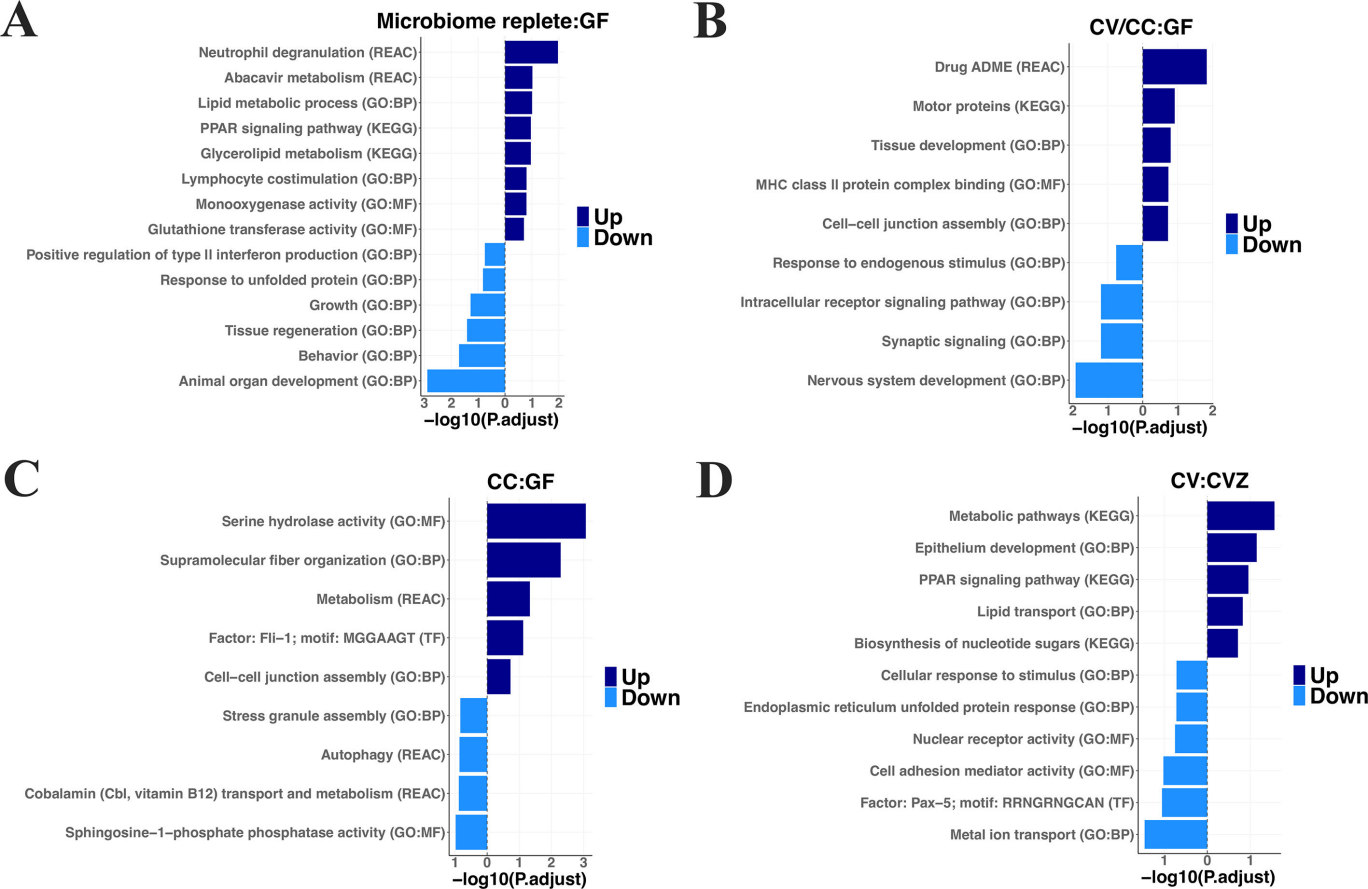
Enriched pathways associated with host gene regulation in response to the microbiome. (**A and B**) Selected pathways enriched among genes regulated in the same direction in (**A**) microbiome-replete versus germ-free (GF) animals and (**B**) CV and CC animals versus GF. (**C and D**) Selected pathways enriched among differentially expressed genes in (**C**) CC versus GF animals and (**D**) CV versus CVZ animals. Bars represent the significance of enrichment (−log_10_ adjusted *P* values). Pathways associated with upregulated genes are indicated in darker blue, while those associated with downregulated genes are shown in lighter blue. The dashed vertical line denotes the baseline for no enrichment. Database sources are indicated in parentheses: Gene Ontology–Biological Process (GO:BP), Gene Ontology–Molecular Function (GO:MF), Kyoto Encyclopedia of Genes and Genomes (KEGG), the Reactome database (REAC), and TRANScription FACtor (TF) database.

To gain deeper insights into how these gene expression differences may impact host physiology, we evaluated gene set enrichment for differentially expressed genes. In total, 763 terms (GO:BP = 522, GO:MF = 194, KEGG = 11, REAC = 29, and TF = 7) were significantly enriched (FDR ≤ 0.2) for up- and downregulated genes in GF animals compared to CVZ, CV, and CC (Fig. 4; Table S6). Consistent with previous work in germ-free zebrafish (65), microbial sensing and immune responses including response to lipopolysaccharide (GO:0032496), innate immune system (R-DRE-168249), neutrophil degranulation (R-DRE-6798695), and T-cell costimulation (GO:0031295) were enriched for genes downregulated in GF zebrafish. Pathways involved in mucosal barrier maintenance, including tight junction assembly (GO:0120192), were similarly reduced. In addition, lipid uptake, transport, and metabolism pathways (GO:0010876, GO:0008289, GO:0006869, and GO:0006629) were downregulated, including PPAR signaling pathway (KEGG:ko03320), a key regulator of lipid metabolism and adipocyte development. In contrast, genes upregulated in GF animals enriched for pathways associated with growth (e.g., developmental growth—GO:0048589 and growth—GO:0040007), cell death (programmed cell death—GO:0012501), and development (e.g., eye development—GO:0001654; central nervous system development—GO:0007417; neuron differentiation—GO:0030182).

Animals that did not undergo GF derivation (CC and CV) uniquely shared upregulated genes enriched in pathways involved in drug absorption, distribution, metabolism, and excretion (ADME) (REAC:R-DRE-9748784), immune function (GO:0007043, GO:0030199, GO:0023026, and GO:0042605), and lipid metabolism (GO:0042577; Fig. 4B). Genes uniquely downregulated in CC compared to GF were enriched in pathways such as autophagy (REAC:R-DRE-9612973 and REAC:R-DRE-1632852), stress granule assembly (GO:0034063), vitamin B12 metabolism (REAC:R-DRE-196741, Fig. 4C) while upregulated genes were enriched in drug ADME (REAC:R-DRE-9748784), and metabolism (REAC:R-DRE-1430728, KEGG:ko00500). Comparing CVZ to CV fish (Fig. 4D), we observed that pathways including epithelium development (GO:0060429), PPAR signaling (KEGG:ko03320), metabolic pathways (KEGG:ko01100), lipid transport (GO:0006869), and cellular response to stimulus (GO:0051716) were enriched for genes upregulated in CV animals (Fig. 4D).

### Microbiome abundances correlate with host transcriptome

**Fig 5:**
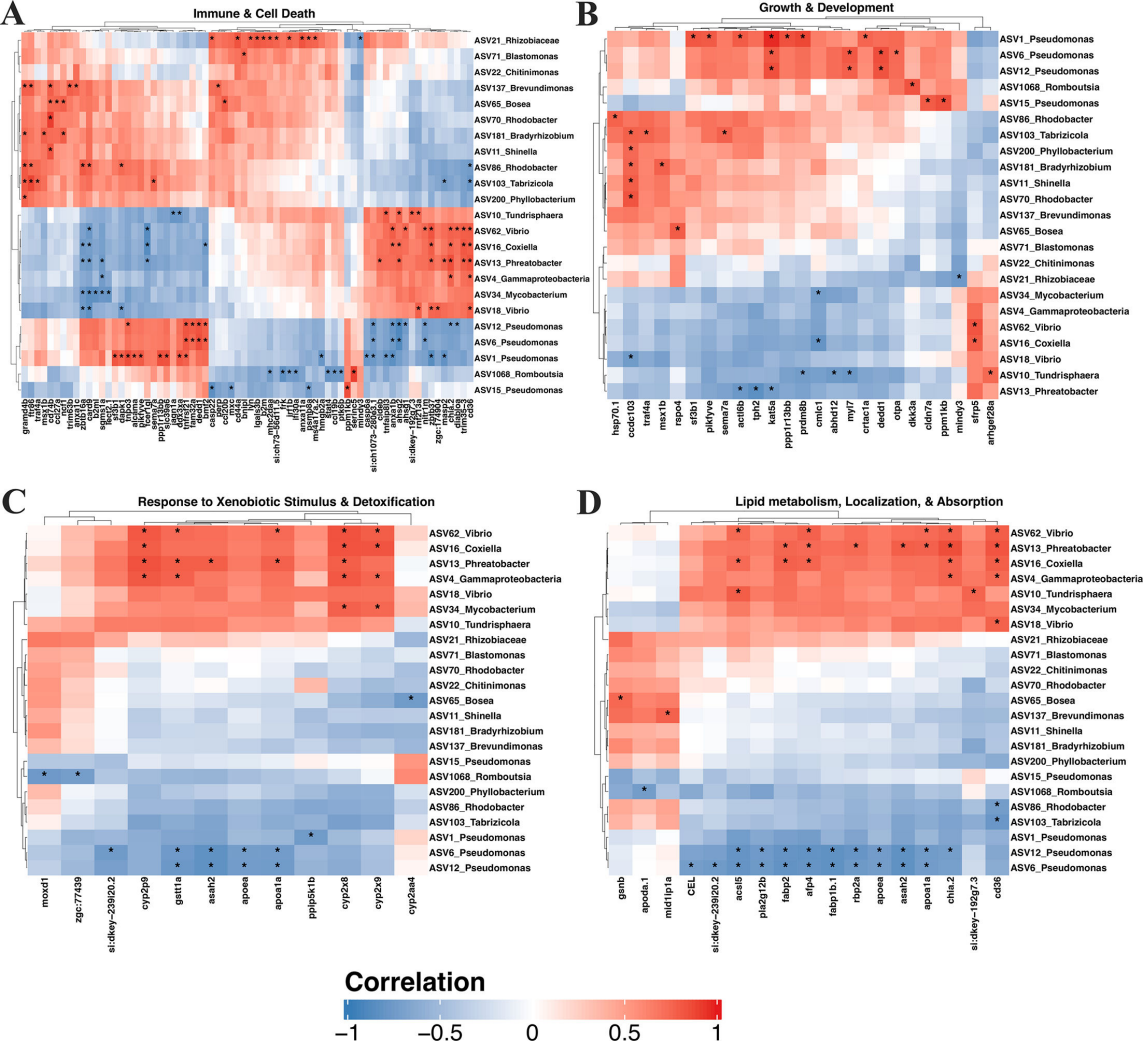
Correlation heatmap of a subset of differentially regulated genes and bacterial taxa. The heatmap illustrates the significant correlations between differentially expressed genes (columns) and bacterial taxa (rows). Colors represent the strength and direction of correlations, with red indicating positive correlations and blue indicating negative correlations. Statistically significant correlations (FDR < 0.1, |*ρ*| > 0.5) are shown with an asterisk. The side dendrograms illustrate hierarchical clustering of genes and taxa. Panels correspond to gene categories: (**A**) Immune & Cell Death; (**B**) Growth & Development; (**C**) Response to Xenobiotic Stimulus & Detoxification; and (**D**) Lipid Metabolism, Localization & Absorption.

To determine if altered gene expression is associated with differences in microbial community composition, we calculated Spearman correlation coefficients between all differentially abundant ASVs and DEGs. We found 2,555 associations (|*ρ*|>0.5, FDR < 0.1) between 24 unique taxa and 1,126 genes (Fig. 5; Table S7). Many of these associations involved abundant commensals of zebrafish larvae including *Pseudomonas* (837), *Vibrio* (285), and *Phreatobacter* (207). These taxa often exhibit patterns of association with host genes that are the inverse of each other. For example, several *Pseudomonas* ASVs negatively correlated with several genes involved in immune function and cell death (Fig. 5A) and lipid metabolic processes (Fig. 5B), while *Vibrio* ASVs showed the opposite trend. Like *Vibrio*, several ASVs belonging to *Tundrisphaera*, *Phreatobacter*, and *Mycobacterium* were positively correlated with critical genes involved in lipid metabolism and uptake including *gstt1a*, *fabp1p.1*, *cyp2p9*, and *cd36*. Expression of several key genes involved in immune functioning and apoptosis (*casp22*, *trim35-3*, *ncf1*, *lyn*, and *stat4*) was also linked to these and other taxa. Similar patterns were seen with microbial associations with genes involved in response to xenobiotics (Fig. 5C) with key regulators of these processes (*cyp2x9*, *cyp2x8*, *cyp2p*, and *gstt1a*) exhibiting distinct patterns of association with *Pseudomonas*, *Vibrio*, and *Phreatobacter* ASVs. Finally, growth, development, and differentiation, including several involved in Notch and Wnt signaling pathways, were linked to numerous taxa (Fig. 5D). Collectively, these data support the hypothesis that a diverse microbiota influence host metabolism, immune stimulation, and response to xenobiotics.

## DISCUSSION

Host-associated microbiota play an important role in development (66, 67), immune function (68–71), metabolism (72, 73), and behavior (74, 75). Gnotobiotic animals offer a tool to explore the mechanistic underpinnings of these associations. The simple methods for germ-free derivation, amenability to high-throughput screening, and transparent early life stages of the zebrafish have led to growing interest in this model. However, constraints on the experimental length and life stages available for investigation have limited its potential. Our work shows that gamma irradiation of pelleted feeds provides a viable method of rearing germ-free zebrafish beyond early larval stages of development. Our approach eliminates barriers for adoption of this model system and has the potential to fast-track future mechanistic studies into host-microbe interactions across developmental stages.

Any long-term method for rearing gnotobiotic zebrafish should minimize impacts on development (39). The impact of irradiated diets was minimal in microbiota-replete animals, with only modest differences in microbial community composition. More significant impacts on microbial community composition were observed between GF control groups (CC, CV, and CVZ), despite all groups receiving the same exposure to parental microbiota. While modest changes in microbiome composition in microbiota-replete animals may be due to stochastic effects or destruction of nutrients caused by irradiation (45, 46), the causes for the more substantial effects in GF controls could have several causes. For example, CC exhibited the most diverse microbiota potentially due to housing and diet differences which are known determinants of diversity in zebrafish and other animal models (76, 77). Previous work has also shown that timing of exposure, microbiome diversity, and initial inoculum composition impact microbial assembly and host development (78–80). It is also possible that residual antimicrobials or disinfectants used in derivation impacted microbial colonization and succession in the CVZ animals (81, 82). Since our data suggest that the resulting communities associate with host gene expression, future work should attempt to minimize the compositional differences between GF experimental controls (i.e., CVZ and CV) and animals reared under standard conditions.

While early larval stages rely primarily on yolk reserves (83) and show minimal size differences, growth deficits become evident in our model as zebrafish have transitioned to exogenous feeding. Specifically, juvenile GF zebrafish exhibited reduced body size and weight. Similar observations have been made in other germ-free animal models, including pigs and mice (84, 85). Germ-free piglets exhibit impaired growth and nutrient digestibility, a phenotype that can be reversed with microbiota transplantation (84). It is well established that host-associated microbiota play a critical role in host nutrient absorption and metabolism (3, 86), so it follows that the reduced weight in our GF animals may reflect defects in these pathways (22, 87). Micronutrients are also closely linked to microbial activity and host physiology. Sterilized rodent diets are vitamin-deficient and cause poor growth, hepatic necrosis, myocardial degeneration, and decreased reproduction (88–91). Vitamin deficiencies similarly impair development and metabolism in zebrafish (92–94), emphasizing the need to evaluate vitamin roles in gnotobiotic zebrafish health. Accordingly, we supplemented germ-free feeds with a vitamin mixture to minimize any potential micronutrient depletion that occurred with gamma irradiation. However, weight, length, and condition factor were still elevated in CC and CV animals. It is unclear if these effects were due to impacts of the GF derivation, microbial action, or dietary factors. It is also unclear how accurately some of these measures reflect pathologies at this developmental timing. For example, the condition factor is primarily used in adults and may not reliably indicate pathology in juvenile zebrafish during early development (95–97). More work is needed to disentangle diet, microbial, and derivation effects on growth and development in germ-free fish to optimize long-term husbandry conditions.

A major limitation of current GF zebrafish models is that animals are not fed, making investigations of microbial interactions with nutrient uptake and metabolism impossible (98). Our protocol enables these investigations, and our results mirror those in mammalian systems. For example, expression of intestinal lipid transporters and fatty-acid-binding protein genes was reduced in GF (5, 7, 65). The decrease in lipid metabolism and localization is consistent with reports in antibiotic-treated zebrafish (99). Genes involved in bile acid homeostasis, including *akr1b1.2*, *ugt2a6*, *apoa1a*, *cyp27a2*, and *cyp3a65*, were also downregulated in GF fish compared to control fish, consistent with microbial modulation of bile acid metabolism in other vertebrates (61, 62). Many of these differences in gene expression link to abundance of specific microbial taxa including commensals of the zebrafish gut. Specifically, ASVs associated with the genus *Pseudomonas* were negatively correlated with *apoa1a*, *fabp2*, *fabp1b.1*, and *acsl5* expression, indicating the possible involvement of these taxa in regulation of fatty acid transport and activation pathways. Consistently, increased abundance of *Pseudomonas* was noted in the smaller CVZ and CV animals compared to CC controls. Whether these observations are cause or consequence of differences in lipid metabolism and the mechanisms that underpin these associations is unclear and warrants further investigation.

Zebrafish are an important model system for liver disease and toxicology (100), as well as the role the microbiome plays in these processes (21, 101). Germ-free animals had significantly lower expression of liver development and hepatocyte differentiation genes. Genes involved in xenobiotic metabolism and sensing were also decreased in GF animals. This finding aligns with prior work suggesting that GF zebrafish are less capable of detoxifying dietary constituents and environmental components (14) and supports a role for microbes in promoting liver development, function, and detoxification as demonstrated in other models (102). Our model provides opportunities for reductionist study of gut-liver-microbiota interactions and their impact on chronic disease and detoxification.

Host-microbiome interactions are crucial for the development and education of immune cells and for maintaining their interactions with microbiota (34, 68). Zebrafish possess a functional innate immune system within days of fertilization, while their adaptive immune system becomes mature and functional within 4–6 weeks of hatch (103). Consistent with findings from other GF animals, GF juvenile zebrafish exhibited altered expression of genes predicted to be involved in innate and adaptive immune functions (68, 104–107). Notably, adaptive immune genes such as *mhc2daa* (108), *cd74b* (109), *b2m* (110), *ccl27a* (111*), slc39a6* (112), *cd44a* (113), *stat4* (114), *psmb8a* (115, 116), *gata3* (117), and *zbtb7b* (118) were downregulated in GF fish, suggesting microbiota-dependent modulation of adaptive immunity. Innate immune genes *il1b* (119), *cd68* (120), *ccl20b* (121), *cpa5* (122), and *icn* (123) were similarly reduced. These changes in innate and adaptive immune genes are consistent with well-established crosstalk between innate and adaptive immunity in vertebrates (124, 125), and may reflect early microbiota-dependent coordination of immune function. CD4^+^ and CD8^+^ T-cell and B-cell marker genes, including cd40 (126), cd40lg (127), cd4-1 (126), cd22 (128), and cd180 (129, 130), were expressed at low abundance relative to most expressed genes. This is consistent with dilution of immune cell-specific signals in bulk RNA-Seq data (131) and means our ability to resolve gene expression heterogeneity in minority cell populations was likely limited in this work. Future application of single-cell/nuclei or spatial transcriptomic approaches could resolve immune cell-specific effects and more precisely define microbiota-driven regulation of immune development and homeostasis (132).

Our study presents a significant first step towards low-cost mechanistic study of host-microbiota interactions. Ongoing experiments in our lab seek to optimize this model even further to rear and spawn adult GF zebrafish for the first time. Slower growth and developmental differences, while common in other GF models, should also be addressed by defining the optimal nutritional parameters, feeding efficiency, microbial colonization timing, and housing conditions for GF and CV zebrafish. Sex has also been shown to influence host-microbiome interactions in zebrafish and other vertebrates (133, 134). However, sex could not be determined by visual inspection at the developmental stage analyzed in this study. Future studies at later developmental stages will incorporate sex as a biological variable to assess potential sex-specific host-microbiome responses. The current approach of manual media changes is no more frequent than cell culture media changes but still may limit scalability; future automation of this step would improve throughput. Long-term culture also presents challenges in maintaining sterility. While our sterility assessments exceeded best practices in germ-free zebrafish husbandry, these were still indirect (i.e., monitoring fish water), meaning very low levels of microbial contamination may have escaped detection. Future investigations should reevaluate best practices in the context of long-term exposure. Finally, although gamma irradiation requires specialized infrastructure, access through shared institutional or commercial facilities makes this approach broadly feasible. Despite these caveats, our approach provides a foundation for future work that can leverage this system to define mechanisms of interactions between host and microbiota across the lifespan.

## Data Availability

All sequencing data used in this article are deposited at the National Center for Biotechnology Information Sequence Read Archive under project numbers PRJNA1338261 and PRJNA1338468, samples SAMN52403128–SAMN52403288 and SAMN52440818–SAMN52440835.

## SUPPLEMENTAL MATERIAL

Supplemental figures msystems.00353-26-s0001.pdf Figures S1 to S3.

Supplemental tables msystems.00353-26-s0002.xlsx Tables S1 to S7.

ASM does not own the copyrights to Supplemental Material that may be linked to, or accessed through, an article. The authors have granted ASM a non-exclusive, world-wide license to publish the Supplemental Material files. Please contact the corresponding author directly for reuse.
