# Supplementary material for: Long-term culture of germ-free zebrafish using gamma-irradiated feeds
Source: mSystems. 2026 Apr 29;11(5):e00353-26. doi: 10.1128/msystems.00353-26 (PMC13185591; doi:10.1128/msystems.00353-26)
Supplement: Supplemental figures — Figures S1 to S3. [file msystems.00353-26-s0001.pdf]

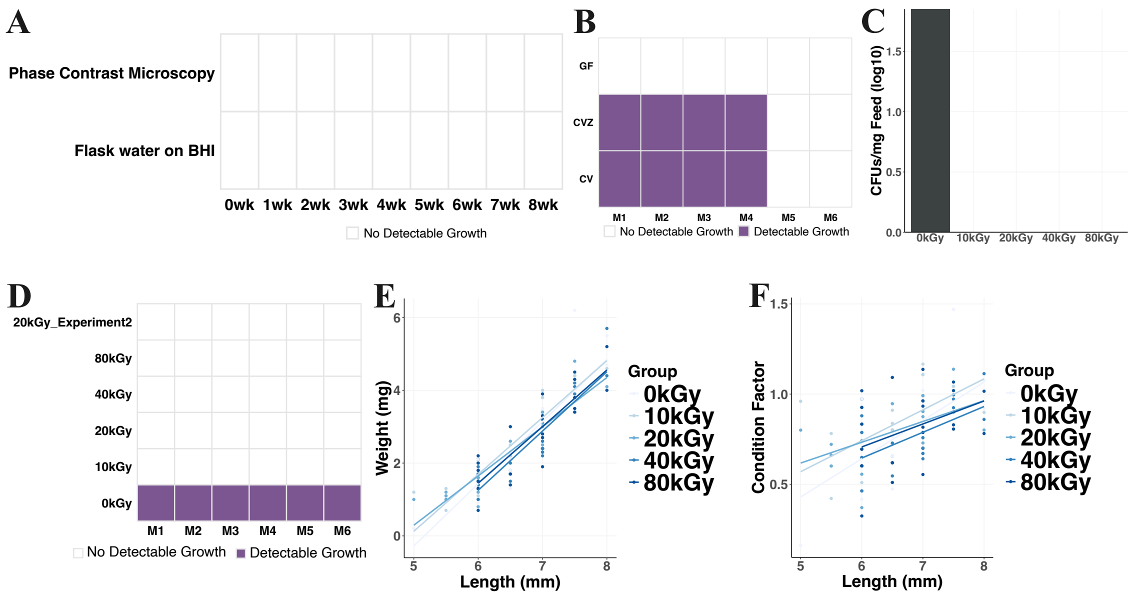

Figure S1: Microbial Load in Fish flasks, Feed, and Size Effects in Zebrafish. A) Growth outcomes of **A)** Flask water cultures from germ-free (GF) flasks grown on BHI under aerobic conditions and assessed by phase contrast microscopy over 8 weeks of the study. **B)** Flask water cultures across the different groups (GF, CVZ, and CV) under six culture conditions. **C)** Bar plot depicting CFUs per mg of feed across different diets. **D)** Microbial cultures following feed irradiation at increasing doses (0-80 kGy; 20 kGy feed used in Experiment 2). Growth was assessed across six culture conditions (M1-M6). In panels A, B, and D, white tiles indicate no detectable growth, while purple tiles indicate detectable growth. Culture conditions were defined as: M1, BHI aerobic; M2, BHI anaerobic; M3, TSA aerobic; M4, TSA anaerobic; M5, SDA aerobic; and M6, SDA anaerobic. **E)** Scatter plot showing the relationship between body length and weight across individual zebrafish fed the different diets. **F)** Scatter plot showing the relationship between body length and condition factor across individual zebrafish fed the different diets.

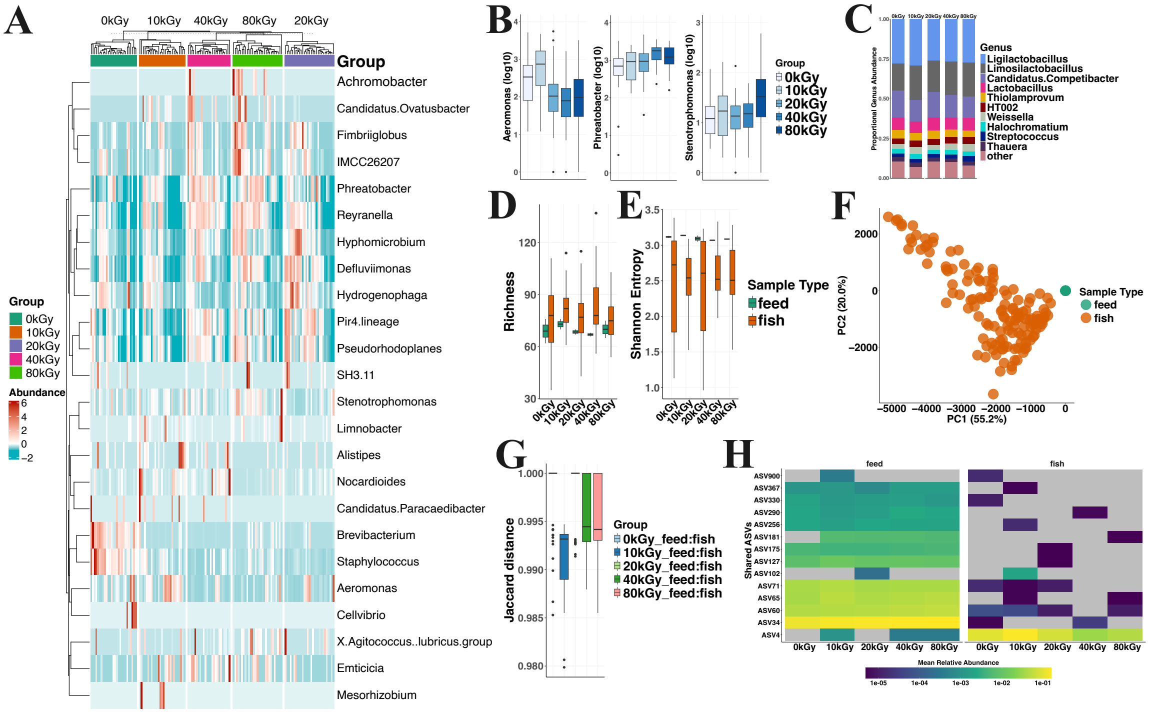

Figure S2: Microbial Diversity, and Community Shifts in Diets and Zebrafish. A) a heatmap of differentially abundant taxa highlighting host-associated shifts in microbial communities. **B)** Boxplots show the log10-transformed abundance of *Aeromonas*, *Phreatobacter*, and *Stenotrophomonas* in fish fed diets irradiated at 0, 10, 20, 40, or 80 kGy. **C)** The relative abundance of the top ten microbial genera in the zebrafish. Boxplots illustrating **D)** microbial richness and **E)** Shannon entropy for both the diets and the zebrafish that consumed the diets. **F)** Principal component analysis (PCA) plot showing the clustering of microbial communities in fish and feed. **G)** Jaccard distances between diet-associated microbial communities and the host-associated microbiota of zebrafish fed those diets. **H)** Tile plot of mean relative abundance for ASVs shared between feed and fish across increasing irradiation doses. ASVs are shown on the y-axis and irradiation dose on the x-axis, with separate panels for feed and fish samples. Color intensity represents mean relative abundance (log10 color scale); grey tiles indicate ASVs not detected in a that group.

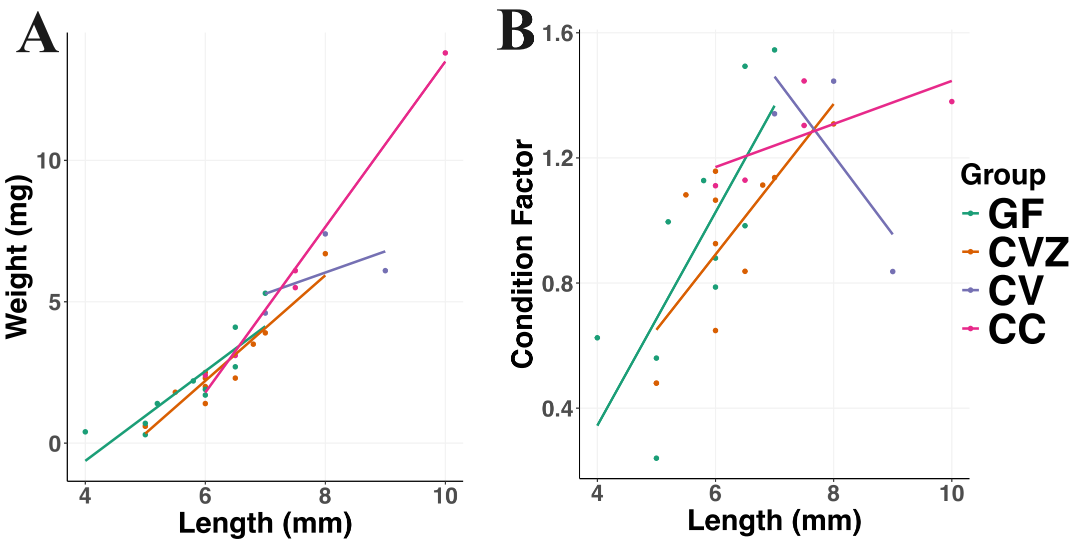

Figure S3: Size Effects in GF, CVZ, CV, and CC Zebrafish. A) Scatter plot showing the relationship between body length and weight across individual zebrafish in the different groups. **B)** Scatter plot showing the relationship between body length and condition factor across individual zebrafish in the different groups.
